# A systematic literature review of the relationship between parenting responses and child post-traumatic stress symptoms

**DOI:** 10.1080/20008066.2022.2156053

**Published:** 2022-12-20

**Authors:** Nimrah Afzal, Siyan Ye, Amy C. Page, David Trickey, Mark D. Lyttle, Rachel M. Hiller, Sarah L. Halligan

**Affiliations:** a University of Bath, Department of Psychology, Bath, United Kingdom; b UK Trauma Council, Anna Freud Centre, London, United Kingdom; c Emergency Department, Bristol Royal Hospital for Children, Bristol, United Kingdom; d Research in Emergency Care Avon Collaborative Hub (REACH), University of the West of England, Bristol, United Kingdom; e Faculty of Brain Sciences, University College London, London, United Kingdom

**Keywords:** PTSD, child trauma, parent–child relationship, post-traumatic stress, parenting, Trastorno de estrés postraumático, trauma infantil, relación padre-hijo, estrés postraumático, crianza de los hijos, PTSD, 儿童创伤, 亲子关系, 创伤后应激, 教养

## Abstract

**Background:** Parents are a key source of support for children exposed to single-incident/acute traumas and can thereby play a potentially significant role in children’s post-trauma psychological adjustment. However, the evidence base examining parental responses to child trauma and child posttraumatic stress symptoms (PTSS) has yielded mixed findings.

**Objective:** We conducted a systematic review examining domains of parental responding in relation to child PTSS outcomes.

**Method:** Studies were included if they (1) assessed children (6-19 years) exposed to a potentially traumatic event, (2) assessed parental responses to a child’s trauma, and (3) quantitatively assessed the relationship between parental responses and child PTSS outcomes. A systematic search of three databases (APAPsycNet, PTSDpubs, and Web of Science) yielded 27 manuscripts.

**Results:** Parental overprotection, trauma communication, avoidance of trauma discussion and of trauma reminders, and distraction were consistently related to child PTSS. There was more limited evidence of a role for trauma-related appraisals, harsh parenting, and positive parenting in influencing child outcomes. Significant limitations to the evidence base were identified, including limited longitudinal evidence, single informant bias and small effect sizes.

**Conclusion:** We conclude that key domains of parental responses could be potential intervention targets, but further research must validate the relationship between these parental responses and child PTSS outcomes.

## HIGHLIGHTS

- Child post-traumatic stress symptoms following acute trauma are consistently related to post-trauma parental overprotection, avoidance of trauma discussion and of trauma reminders, and promotion of distraction from trauma-related thoughts and stimuli.
- The findings from this review provide a potential rationale for targeting these parental domains in clinical interventions addressing children’s post-traumatic stress symptoms.
- Future research is needed to validate the longitudinal relationship between parental response domains following children’s traumatic exposure and child post-traumatic stress symptoms.

## Abstract (es)

**Antecedentes:** Los padres son una fuente clave de apoyo para los niños expuestos a traumas agudos/ de un solo incidente y, por lo tanto, pueden desempeñar un papel potencialmente significativo en el ajuste psicológico posterior al trauma de los niños. Sin embargo, la base de la evidencia que examina las respuestas de los padres al trauma infantil y a los síntomas de estrés postraumático infantil (PTSS) ha dado resultados mixtos.

**Objetivo:** Llevamos a cabo una revisión sistemática examinando los dominios de la respuesta parental en relación con los resultados del PTSS infantil.

**Método:** Se incluyeron estudios si estos:(1) evaluaron a los niños (6-19 años) expuestos a un evento potencialmente traumático, (2) evaluaron las respuestas de los padres al trauma de un niño y (3) evaluaron cuantitativamente la relación entre las respuestas de los padres y los resultados del PTSS del niño. Una búsqueda sistemática de tres bases de datos (APAPsycNet, PTSDpubs y Web of Science) produjo 27 manuscritos.

**Resultados:** La sobreprotección parental, la comunicación sobre el trauma, la evitación de la discusión sobre el trauma y los recordatorios de trauma, y la distracción, se relacionaron consistentemente con el PTSS infantil. Hubo evidencia más limitada del rol de las evaluaciones relacionadas con el trauma, la crianza dura y la crianza positiva, sobre la influencia de los resultados infantiles. Se identificaron limitaciones significativas a la base de evidencia, incluida evidencia longitudinal limitada, sesgo de un solo informante y un pequeño tamaño de efecto.

**Conclusión:** Concluimos que los dominios clave de las respuestas parentales podrían ser objetivos de potencial intervención, pero más investigaciones deben validar la relación entre estas respuestas parentales y los resultados del PTSS infantil.

## Abstract (zh)

**背景：**父母是单一事件/急性创伤暴露的儿童的主要支持来源，因此会在儿童创伤后心理调节中发挥潜在重要作用。然而，考查了父母对儿童创伤和儿童创伤后应激症状 (PTSS) 反应的证据基础结果混杂。

**目的：**我们进行了一项系统综述，考查与儿童 PTSS 结果相关的父母反应领域。

**方法：**如果研究（1）评估了潜在创伤事件暴露儿童（6-19 岁），(2) 评估了父母对儿童创伤的反应，以及 (3) 定量评估了父母反应与儿童 PTSS 之间的关系，则纳入研究结果。对三个数据库（APAPsycNet、PTSDpubs和 Web of Science）的系统搜索获得了 27 份稿件。

**结果：**父母的过度保护、创伤沟通、避免讨论创伤和创伤提示物，以及分心与儿童 PTSS 始终相关。有关创伤相关评估、严苛教养和积极教养对儿童结果的影响的证据更为有限。确定了证据基础的重大局限性，包括有限的纵向证据、单一信息提供者偏差和小效应量。

**结论：**我们得出结论，父母反应的关键领域可能是潜在的干预目标，但进一步的研究必须验证这些父母反应与儿童 PTSS 结果之间的关系。

## Introduction

It is estimated that at least one third of individuals will experience a traumatic event during childhood, with the prevalence varying widely across different populations and types of trauma exposures (Lewis et al., 2019). For example, in the USA based National Comorbidity Survey Replication, 36% of participants reported trauma occurring before 11 years, including interpersonal violence (11%), serious accidents (18%), and witnessing or hearing about a trauma happening to a loved one (15%) (Carliner et al., 2016). Single-incident, acute traumatic events (e.g. road traffic accident, severe injury, assault, natural disasters) have received less research attention than complex or chronic traumatic experiences (e.g. maltreatment, abuse, and neglect) (Adler-Nevo & Manassis, 2005). Although chronic interpersonal traumas are associated with higher risk of mental health problems (Alisic et al., 2014), single-incident traumas can affect large numbers of children and lead to PTSD and other mental disorders (Bauer et al., 2022). For example, between 2–3 million children and adolescents aged 0–17 years old have attended emergency departments between 2020–21 in the UK (NHS Digital, 2021) and common reasons for admissions include injuries from events such as motor vehicle accidents, burns, and falls (Public Health England, 2018). Given the challenges of studying parental support in the context of complex/chronic trauma, which frequently involves family members, and the large numbers of children affected by single-incident events, the current review is focused on parenting and PTSD in the context of acute child trauma.

Following exposure to single-incident trauma, a meta-analysis of longitudinal studies found that 21% of children met symptom criteria for PTSD at 1-month post-trauma, with 15% meeting criteria for at least 3 months (Hiller et al., 2016). Symptoms of PTSD include trauma-related intrusions (e.g. distressing memories of the trauma, nightmares), avoidance of trauma reminders, negative alterations in cognitions and mood (e.g. negative affect, negative changes in beliefs about the world/other people) and marked alterations in arousal and reactivity (e.g. irritability, hypervigilance) (American Psychiatric Association, 2013). While only a subset of children exposed to trauma will be given a clinical diagnosis of PTSD, many more will experience posttraumatic stress symptoms (PTSS) ranging from mild to severe.

Providing post-trauma support to children and young people is paramount to immediate and long-term recovery. If left unaddressed, childhood trauma exposure and PTSD are associated with long-term consequences, including increased risk of major depressive episodes, alcohol dependence and suicide attempts (Lewis et al., 2019). However, only a minority of young people with PTSD access mental health support (Lewis et al., 2019). Reasons for this include a lack of awareness of mental health services, a lack of culturally sensitive treatments, and living in an area of low socioeconomic status which is associated with factors including a mistrust of the healthcare system, stigma, and a lack of treatment options (Hodgkinson et al., 2017). Given limited treatment access, there is opportunity for novel approaches to addressing support for trauma exposed children. One potential avenue is to focus on the role of parents in children’s post-trauma psychological adjustment (Bokszczanin, 2008).

Marsac et al.’s (2014a) model of paediatric PTSS following acute injuries/medical events highlights the significance of parental coping assistance for the child, parenting styles, and the parent’s own PTSS in relation to the child’s trauma. Existing evidence has highlighted associations between parental PTSD and parenting domains including increased parenting stress, increased and inconsistent discipline, and hostility (Christie et al., 2019), including PTSD related to the child’s trauma (e.g. Wilcoxon et al., 2021). In addition, a growing body of research has examined associations between parental responses to child trauma and subsequent child PTSS, specifically in single-incident, acute child traumas, with previous reviews having synthesised specific components of this literature. Thus, Cobham et al. (2016) provided insight into the role of the anxious parenting, maladaptive trauma discussion, and family conflict in children exposed to disaster. In addition, Williamson et al. (2017) examined broad negative and positive parenting domains (e.g. sensitivity, hostility) in relation to a range of trauma types, and Wise & Delahanty (2017) examined the same parenting targets specifically for injured children. An updated review that covers all key parenting domains studied and provides a synthesis that is generalisable across a range of single-incident trauma types is warranted, particularly to identify specific, modifiable intervention targets that could be addressed in the paediatric post-trauma period (Trickey et al., 2012).

We conducted a systematic review and synthesis of evidence examining parental responding in relation to children’s PTSS. We included single-incident, acute trauma exposures (e.g. accidental injuries, terror attacks, and natural disasters) and investigated trauma-specific as well as more general parental responses, in order to provide an up-to-date and comprehensive examination of the literature. Key domains of parental responses following child traumatic exposure that were examined included: (a) parenting styles, such as hostile or overprotective parenting, (b) parents’ own negative appraisals following the child’s trauma, and (c) coping strategies, including cognitive or behavioural avoidance of trauma material. As there is evidence that a child’s exposure to trauma can trigger changes in parenting behaviour (Cobham & McDermott, 2014; Salmon & Bryant, 2002) we focused only on studies that examined parental responding subsequent to the child’s trauma.

## Methods

### Search strategy

**Figure 1.**
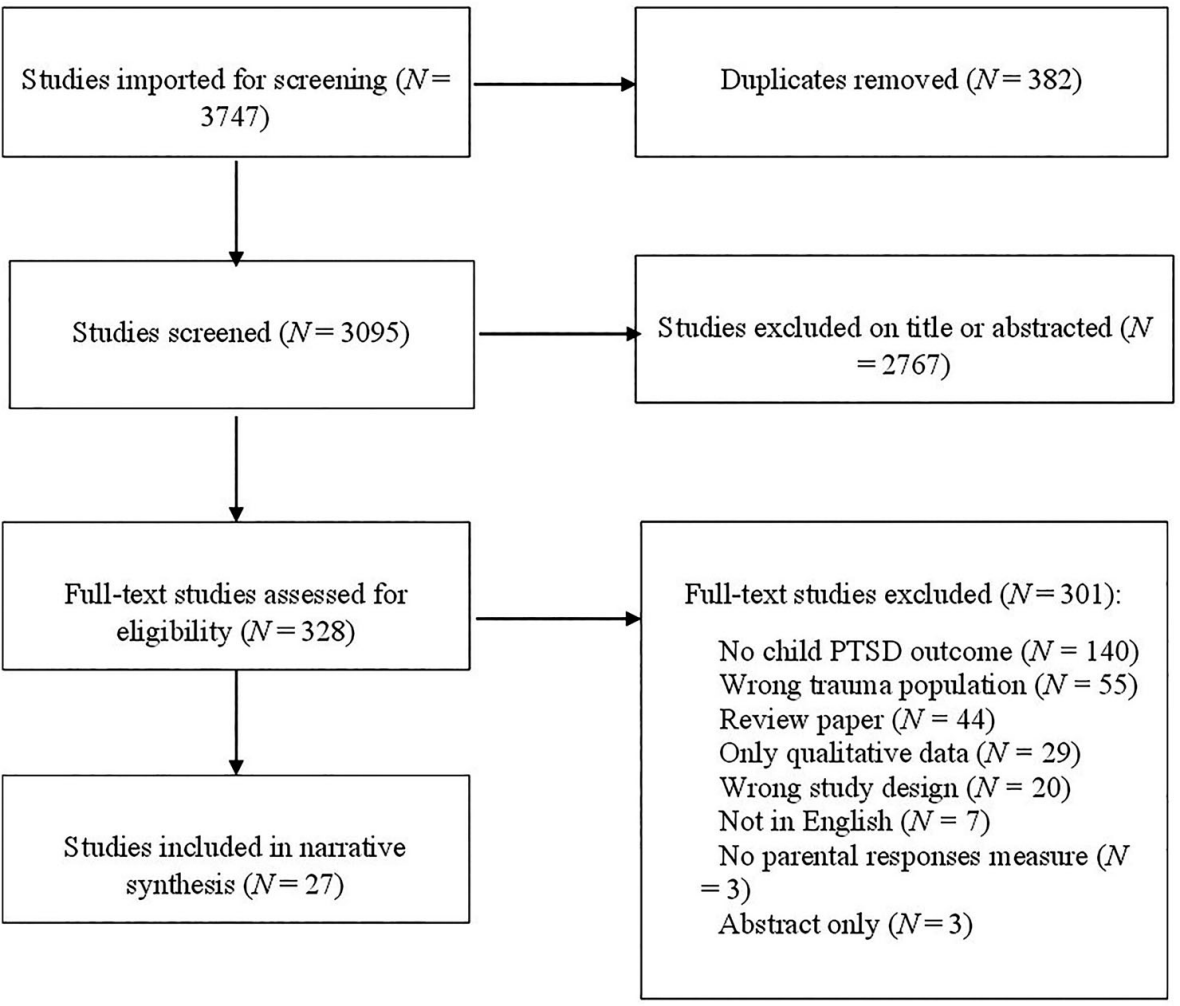
PRISMA Flow Diagram of study Screening and inclusion.

The review was pre-registered on PROSPERO (registration number: CRD42021216535). We conducted the review according to Preferred Reporting Items for Systematic Reviews and Meta-Analyses (PRISMA) guidelines and a PRISMA flow chart (Moher et al., 2009) (Figure 1).

The search strategy was designed in close consultation with a university librarian. The literature search was carried out using electronic databases: APAPsycNet (PsycInfo, PsychArticles, PsycExtra), PTSDpubs and Web of Science. APAPsychNet and Web of Science were selected for the breadth of papers indexed, and PTSDpubs was selected for its specific indexing of PTSD research. The search terms were based upon previous reviews which the current paper aimed to update (Cobham & McDermott 2014; Williamson et al., 2017): ‘trauma*’, OR ‘post-trauma*’, OR ‘PTSD’, OR ‘PTSS’, AND ‘child*’, OR ‘adolescen*’, AND ‘parent* beh*’ OR ‘famil*. The search was limited to papers published between 1980 (as this is when PTSD was first defined by the DSM) and January 2021. The search was conducted by the first author (NA) and papers were imported into COVIDENCE. Here, duplicate papers were removed, and 3023 papers were screened. References lists were manually searched to screen relevant titles not captured in the initial search.

### Study selection

**Selection criteria.** Studies were included if they: (a) assessed children who experienced a traumatic event consistent with the DSM/ICD diagnostic criteria for PTSD, (b) assessed child PTSD/PTSS using a validated measure, (c) examined parenting domains and/or parental post-trauma responses following child trauma exposure in relation to children’s PTSS, (d) had a sample with average age between 6–19 years (or range where average age was not reported) as the DSM-5 has separate criteria for PTSD diagnosis for children below 6 years old (McLaughlin et al., 2018), (e) comprised of peer reviewed original research, written in English.

Studies were excluded if: (a) the child trauma was deliberately perpetrated/ inflicted by the parent (e.g. child abuse/neglect), (b) child PTSS/PTSD was investigated in the context of a primary co-morbid physical and/or mental health condition (e.g. traumatic brain injury, cancer or psychosis), (c) the child trauma was still ongoing, (d) they investigated parental relationship quality/attachment only prior to the child’s traumatic exposure, (e) they were single case studies, intervention studies or reported only qualitative data.

**Screening process.** The lead author screened the title and abstracts of all studies (*N* = 3023) and a second reviewer (AP) screened 50% of the papers, with 96% agreement. Any disagreements regarding the eligibility of studies were discussed with SLH. From this initial screen, 328 papers were retained for a full text screening. Authors NA and SY independently screened all papers, with 87% agreement. All disagreements were resolved at a consensus meeting with author SLH. Reasons for study exclusion were recorded, and common reasons for exclusions included no quantitative measure of child PTSD/PTSS and children experiencing chronic/ongoing trauma or trauma where the parent is the perpetrator. This resulted in 27 papers (Figure 1).

### Data collection and quality assessment

We extracted the following data: (a) bibliographic information, (b) study design, (c) sample size (children and parents), (d) child age range and mean, (e) parent age range and mean, (f) child sex, (g) parent sex, (h) trauma type, (i) parenting response measure, (j) parenting response domain (e.g. overprotection or avoidance), (k) parenting informant, (l) child PTSS informant, (m) child PTSS measure, (n) child PTSS (*M, SD*), and (o) association between parental response and child PTSS (measure of association, significance, and *SD*)

Two authors (NA and SY) independently extracted data into Covidence and assessed the quality of the included studies using the Risk of Bias Assessment Tool for Nonrandomized Studies with 80% agreement (RoBANS) (Kim et al., 2013). Final decisions were reached through a consensus meeting with author SLH.

The RoBANS is a valid tool for evaluating cohort, case–control, and before-after studies. The RoBANS was chosen for its applicability across these different study designs and evaluation across key domains relevant to etiological studies. The original tool assesses the risk of bias across six domains, but an additional domain for ‘study design’ was included for the present review: selection of participants, confounding variables, measurement of exposure, study design, blinding of outcome assessments, incomplete outcome data, and selective outcome reporting. Each domain is scored as ‘low’, ‘high’ or ‘unclear’ risk of bias. Outcomes of the quality assessment are reported in the results section and Supplementary Material B.

## Results

### Overview of included studies

Figure 1 provides a PRISMA flow diagram of the final 27 papers (*N *= 22,425, children and parents). Eighteen papers presented cross-sectional studies, and nine were longitudinal. Children’s traumatic exposure included terror/war (*k* = 11), natural disasters (*k* = 7), acute injuries (*k* = 2), motor vehicle accidents (*k* = 1), bereavement (*k *= 1), and mixed trauma types (*k *= 5). Study summaries are provided in Table 1, with a comprehensive description in Supplementary Material A.

**Table 1.** Summary of Included Studies.

Reference	Trauma type	Sample size	Child age in years, *M* (SD)	Mothers (%)	PTSS Informant	Parenting Informant	Parental response
Bokszczanin (2008)	Natural disaster (flood)	533 children	16.0 (2.50)	n/a	Child	Child	Positive parenting, overprotection
Carpenter et al. (2017)	Terror/bombing	460 dyads	11.8 (3.8)	81%	Parent	Parent	Trauma communication
Cobham & McDermott (2014)	Natural disaster (minicylone)	874 dyads	9.71 (1.16)	Not reported	Child	Parent	Overprotection, trauma communication, parent appraisals
Cohen & Eid (2007)	Terror/war-related	346 children	Mean not reported, 13–15 years old	100%	Child	Child	Overprotection
Dekel & Solomon (2016)	War	2858 children	13.5 (0.65)	n/a	Child	Child	Positive parenting, overprotection
Dubow et al. (2012)	Terror/political violence	1501 parent-child dyads	Not reported, aged 8–14 years old	84%	Child	Parent	Positive parenting
El-Khodary & Samara (2019)	War	1029 children	13.7 (1.36)	n/a	Child	Child	Positive parenting, harsh parenting
Felix et al. (2020)	Natural disaster (flood)	485 parent-children dyads	13.8 (2.56)	69%	Child	Both	Trauma communication
Garfin et al. (2014)	Natural disaster (earthquake)	117 children	7.59 (0.65)	n/a	Child	Child	Caregiver-child conflict, trauma communication
Gil-Rivas & Kilmer (2013)	Natural disaster (Hurricane Katrina)	Time 1: 68 caregiver-child dyads Time 2: 53 caregiver-child dyads	8.5 (1.1)	88%	Child	Both	Positive parenting, coping strategies, trauma communication, caregiver-child conflict
Goddard et al. (2019)	Various	66 parent-child dyads	13.5 (2.7)	97%	Child	Both	Harsh parenting
Hendricks & Bornstein (2007)	Terror/9/11	97 mother-child dyads	13.9 (0.26)	100%	Child	Child	Caregiver-child conflict, harsh parenting
Hiller et al. (2018)	Various	132 parent-child dyads	9.87 (1.8)	90%	Child	Parent	Overprotection, positive parenting, parent appraisals, coping strategies
Kelley et al. (2010)	Natural disaster (Hurricane Katrina)	381 parent-child dyads	12.0 (2.0)	99%	Child	Parent	Coping strategies, harsh parenting
Lavi et al. (2016)	War	65 children	12.3 (1.37)	n/a	Child	Child	Trauma communication
Marsac et al. (2014)	Motor vehicle accident	243 parent-child dyads	11.3 (2.5)	Not reported	Child	Both	Positive parenting, coping strategies
Marsac et al. (2013)	Acute injury	82 parent-child dyads	12.1 (2.7)	82%	Child	Both	Positive parenting, coping strategies
Meiser-Stedman et al. (2006)	Acute injury	66 parent-child dyads	13.8 (1.9)	97%	Child	Parent	Caregiver-child conflict, overprotection
Morris et al. (2016)	Death	62 children, 88 parents	13.0 (3.59)	68%	Child	Child	Positive parenting
Prinstein et al. (1996)	Natural disaster (hurricane)	506 children	M(SD) not reported; 3rd grade = 32%, 4th grade = 31%, 5th grade = 37%	n/a	Child	Child	Trauma communication, positive parenting, coping strategies
Punamäki et al. (2015)	War	240 children, 170 parents	11.4 (0.57)	Not reported	Child	Parent	Harsh parenting
Punamäki et al. (2001)	War	86 children	14.0 (0.79)	n/a	Child	Child	Positive parenting, harsh parenting
Thabet et al. (2009)	War	412 children	13.7 (1.05)	n/a	Child	Child	Positive parenting
Trentacosta et al. (2016)	War	211 children	12.8 (3.17)	n/a	Child	Child	Positive parenting
Valentino et al. (2010)	Various	91 children, 100 parents	12.1 (2.9)	89%	Both	Both	Positive parenting, harsh parenting
Williamson et al. (2018)	Various	365 parent-child dyads	8.2 (3.4)	90%	Parent	Parent	Parent appraisals, coping strategies, positive parenting, overprotection
Zhai et al. (2015)	Various	5765 children	12.5 (1.54)	n/a	Child	Child	Harsh parenting

#### Study samples

Young people ranged from 2–19 years old (*M =* 7.59 – 16.0 years; information missing = 4), with male and female children well represented across studies. Parents/caregivers were assessed in 17 studies, of which two studies had female/mother-only samples and the remainder recruited mostly mothers/female caregivers (≥ 50%) (*k *= 12; information missing = 3), with samples aged 25–63 years old (*M = *37.6 - 44.9 years; missing *k* = 10).

#### Measurement of PTSS

Child PTSS was measured using child-reports (*k = *24), both parent and child reports (*k* = 1), or parent reports (*k* = 2). The majority of the measures were self-report (*k *= 25*),* while a minority used structured clinical interviews (*k *= 2). The most commonly used measures were variations of the UCLA PTSD Reaction Index (Pynoos et al., 1987) (*k *= 8) and the Children’s Impact of Events Scale (Dyregrov & Yule, 1995) (*k *= 3).

#### Measurement of parental responses

Parental responses were reported across six main domains: overprotection, trauma communication, parental promotion of distraction and avoidance, parental trauma-related appraisals, harsh parenting, and positive parenting (see Table 1 study characteristics and Table 2 for definitions of domains). The majority of studies relied on child reports of parental responses (*k *= 13), eight studies obtained parent’s self-reports, and six studies collected separate reports from both children and parents. The measures used were highly varied.

**Table 2.** Parental Response Domain Definitions and Study Design Characteristic*s.*

Parenting Domain	Characteristics	Example of Responses	Number of Studies	Range of average age of children (in years)	Number of longitudinal studies	Number of studies who recruited children and parents
Overprotection	Intense levels of protection High levels of physical and social contact Controlling behaviours to inhibit child’s independence	Controlling parenting Inhibition of autonomy Emotional overinvolvement	7	8.2–19.6	3	3
Trauma communication	Communication with child regarding the child’s traumatic exposure	Avoidance of discussing child’s traumatic experience Co-rumination of trauma experience	6	7.6–12.3	2	3
Parental promotion of distraction and avoidance	Parents promoting maladaptive coping strategies to deal with children’s trauma	Behavioural distraction from trauma reminders Cognitive avoidance of trauma material Parental encouragement of emotional processing of trauma reminders, e.g. through conversation, or trauma-related play	6	8.5–12.1	5	6
Trauma related parental appraisals	Parents’ own appraisals relating to children’s traumatic exposure	Perceiving that the child was permanently damaged Heightened sense of future danger Preoccupation with child’s vulnerability Ruminating about child’s trauma	3	8.2–13.0	2	3
Harsh parenting	Authoritarian parenting characterised by control and discipline	Psychological control Behavioural control Rejection Punishment Hostility	7	11.4–14.0	3	5
Positive parenting	Parenting practices that provide social and emotional support for the child	Emotional warmth Reinstitution of roles and routines	15	8.2–16.0	6	8

### Quality assessment

**Figure 2.**
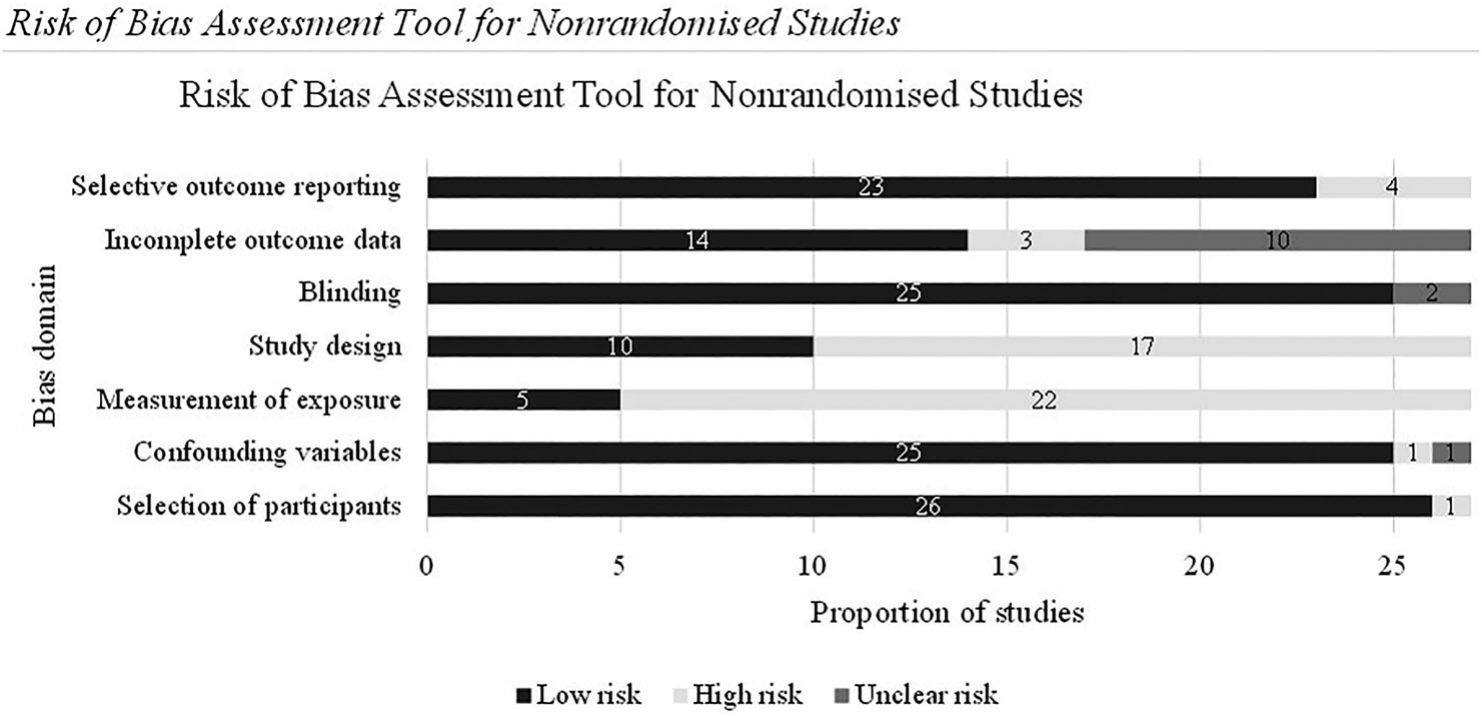
Risk of Bias Assessment Tool for Nonrandomised studies.

The risk of bias assessment using the RoBANS is summarised in Figure 2 and Supplemental Material B. Only one study was judged as low risk of bias across all the domains (Tutus et al., 2019).

The measurement of exposure domain had the highest risk of bias as most studies relied on self-report and a single informant for both child PTSS and parental responses. Many studies also had a high risk of bias in the study design domain as they used a cross-sectional study design, limiting conclusions regarding the directionality of associations between parental responses and child PTSS outcomes. Most studies scored ‘low’ in the risk of bias for confounding variables (*k *= 25) as key potential confounders (e.g. child sex, time since trauma) were controlled for analyses. The overall risk of bias for completeness of outcome data was variable: 14 studies had a ‘low’ risk of bias, due to the use of multiple imputation methods or appropriate analyses to conclude that data were missing at random, with the remainder being ‘unclear’ (*k *= 10) or ‘high’ (*k* = 3, significant missing data which was not addressed in analyses).

## Results

### Overprotection and overcontrol

Seven studies explored parental overprotection (Table 1) which refers to excessive monitoring of and physical/social contact with children (Table 2). There was consistent evidence for a cross-sectional relationship between parental overprotection and child PTSS. Bokszczanin (2008) (*N* = 533) reported an association between adolescent-reported parental overprotection and PTSS (*r* = .40) following flood exposure. Similarly, Meiser-Stedman et al. (2006) found that higher parent-reported maternal overprotection at 6 months post-trauma was cross-sectionally associated with child-reported PTSD diagnosis among children presenting to hospital emergency departments (EDs), but not with PTSS (*ρ* = .38) (*N* = 66). Similarly, Williamson et al. (2018) measured overprotection in 365 children across five different samples exposed to mixed traumas, capturing parent self-reports of both general tendencies towards overprotection (Parental Overprotection Scale, POS; Edwards et al., 2010) and trauma-specific overprotection (e.g. guarding against a similar event happening again; Parental Trauma Response Questionnaire, PTRQ). Parent-reported child PTSS were cross-sectionally associated with higher levels of both trauma specific (PTRQ *r* = .18) and general parental overprotection (POS *r* = .38). By contrast, Hiller et al. (2018) conducted a longitudinal analysis of 132 parent–child dyads recruited via EDs. They found that parent-reported trauma-specific overprotection (PTRQ) predicted higher child-reported PTSS at 1 month (*β* = .18) and 6 months (*β* = .18) post-trauma. However equivalent effects did not emerge for parent-reported general overprotection (POS) or observed parental anxious overinvolvement.

Three cross-sectional studies specifically explored parental control as a form of overprotection, including strategies such as limiting autonomy/child independence. An association with child PTSS was consistently demonstrated. Cohen and Eid (2007) found a positive association between children’s reports of parents forbidding them from going out and self-reported PTSS (*r* = .30; *N* = 346) following war exposure. Dekel and Solomon (2016) also investigated children exposed to war (*N**** ***= 2858) and found a positive association between child-reported maternal control and child-reported PTSS (*r* = .17). Finally, in a sample of children exposed to disasters (*N* = 874), parent-reported inhibition of autonomy (OR_adj_ = 2.44) was associated with an increased risk of child-reported PTSD (OR_adj _= 2.09) (Cobham & McDermott, 2014).

Overall, these studies suggest that parental overprotection is positively associated with child PTSS, with effect sizes ranging from small to medium. However, longitudinal evidence of predictive effects is limited to a single study, and effects tended to be larger/more robust when relying on cross-sectional evidence and/or single informants.

### Trauma communication

Six studies explored the role of parental communication regarding the child’s traumatic experience (Table 1), focusing on the extent to which parents engaged with discussing trauma material. The majority of these studies examined children exposed to natural disasters and found consistent cross-sectional associations. A study of 485 children exposed to floods found that parent-reported (but not child-reported) trauma-related topic avoidance (*r *= .42) and both parent-reported (*r* = .44) and child-reported (*r* = .44) topic co-rumination were associated with greater child-reported PTSS levels (Felix et al., 2020). Similarly, Garfin et al. (2014) (*N *= 117) found child-reported caregiver unavailability to discuss the trauma was cross-sectionally associated with child-reported PTSS following flood exposure (*β* = .21). Finally, Cobham and McDermott (2014) found that parent-reported avoidance of trauma discussion was associated with children-reported PTSD risk (*χ²* = 7.93, no effect size available) in cyclone exposed children (*N *= 874).

These cross-sectional patterns have also been found in samples where children were exposed to terror attacks and war. A study of 460 children exposed to a bombing found a positive association between parent-reported discussion avoidance and parent-reported child PTSS (*β* = .11) (Carpenter et al., 2017). By contrast, in a study of 65 children exposed to war in Gaza, trauma discussion in terms of child-reported parental intermediation regarding news broadcasting was unrelated to child-reported PTSS (Lavi et al., 2016).

There is limited longitudinal evidence regarding the relationship between parent avoidance of trauma discussion and child PTSS. Gil-Rivas and Kilmer (2013) explored the role of caregiver unavailability for trauma discussion following Hurricane Katrina (*N *= 68) and identified a cross-sectional association with child PTSS (both child-reported; *r *= .37). However, a longitudinal association between caregiver unavailability and child PTSS a year later was not found.

Overall, the evidence is relatively consistent in identifying cross-sectional associations between parental avoidance of discussing the trauma and child PTSS. However, there is no longitudinal evidence of the same association and many studies have relied on single informants, which limits the validity and directionality of the association between parental avoidance of trauma discussion and child PTSS. Moreover, all studies identified examined community level exposures (disasters or bombings) which may have unique characteristics in terms of support, therefore evidence may not extrapolate to other trauma populations.

### Parental promotion of distraction and avoidance

Six studies explored parental promotion of distraction-based coping (e.g. with anxiety and fear) and avoidance of stress-related emotions and trauma reminders among children (see Table 1 for specific study details). There was consistent evidence for a cross-sectional relationship between these strategies and child PTSS. Gil-Rivas and Kilmer (2013) reported that both parent-reported promotion of distraction (*r *= .40) and avoidance (*r* = .41) were associated with child-reported PTSS in a sample of children following a natural disaster (*N* = 68). Prinstein et al. (1996) only explored the parent’s own use of distraction among children exposed to hurricanes (*N *= 506) and found that children who reported higher PTSD severity were more likely to report that their parents used distraction as coping assistance (*F *= 19.54). Williamson et al. (2018) explored parent-reported promotion of avoidance in 365 children exposed to various trauma types and found positive relationships with parent-reported child PTSS for both behavioural (*r* = .41) and cognitive avoidance promotion (*r* = .26) measured via the PTRQ.

By contrast, longitudinal evidence is mixed. Marsac et al. (2013) failed to find longitudinal associations between early parent-reported distraction and later child-reported PTSS in 82 children with acute injuries. A second study of children exposed to motor vehicle accidents (*N =* 243) found that children who reported clinically significant PTSS one-month post-trauma were more likely to report their parents using distraction to help them cope six months post-trauma, compared to children without clinically significant PTSS *(χ*²_2 _= 6.03, no effect size available) (Marsac et al., 2014b). However, the direction of this relationship does not provide evidence for the influence of parental use of distraction on child PTSS levels. Finally, Hiller et al. (2018) found that both parental promotion of cognitive (*β* = .19) and behavioural avoidance (*β* = .27) at 1-month post-trauma were associated with child PTSS 6 months post-trauma in 132 children exposed to various trauma types. However, only the effect for behavioural avoidance promotion was retained when controlling for initial child PTSS (*β* = .17).

Overall, these studies suggest that parental promotion of distraction and avoidance, are cross-sectionally associated with child PTSS. However, the only longitudinal association identified was for parental avoidance promotion.

### Trauma-Related parental appraisals

Three studies explored trauma-related parental appraisals (Table 1), comprising parents’ maladaptive cognitions of the child’s traumatic experience and psychological adjustment (see Table 2 for details). Two studies identified cross-sectional relationships between parental appraisals and child PTSS, although the type of appraisals explored varied across studies. Cobham and McDermott (2014) found that parent-reported rumination about children’s exposure to a mini-cylone (*N* = 874) was associated with increased risk of child-reported PTSD (*OR_ad_*_j_**_ _= **1.18). Williamson et al. (2019) (*N *= 365 children with mixed traumas) found associations between parent-reported appraisals of the child being permanently changed (*r *= .51), preoccupation with the child’s vulnerability (*r* = .48) and self-directed blame for child’s trauma (*r *= .32) on the PTRQ with parent-reported child PTSS. Moreover, in the only longitudinal study in this area, Hiller et al. (2018) found child-reported PTSS 6 months post-trauma was predicted by 1-month parent-reported appraisals of perceiving their child as permanently damaged (*β* = .43), preoccupation with their child’s vulnerability (*β* = .32) self-directed blame (*β* = .25), and negative appraisals during a trauma narrative task (*β* = .21). These longitudinal effects were attenuated but still present even when controlling for children’s initial PTSS.

Overall, there is consistent evidence that parental negative appraisals following their child’s traumatic exposure are associated with child PTSS, with both longitudinal and cross-sectional associations. However, as the body of evidence is extremely small, it is difficult to draw conclusions about the effectiveness of targeting parent appraisals to reduce child PTSS.

### Harsh parenting

Seven studies explored the relationship between harsh parenting and child PTSS (Table 1), with harsh parenting encompassing inconsistent and harsh discipline, rejection/hostility, and psychological control (see Table 2 for further details). Several cross-sectional studies of children exposed to mixed trauma types found evidence of associations between elements of harsh parenting and child PTSD, but only for child reported harsh parenting. A study of 2292 adolescents found that authoritarian parenting, characterised by harsh discipline and punishment, was positively correlated with adolescent PTSS (both adolescent-reported: *r* = .26). Conversely, authoritative parenting, characterised by positive reinforcement, was negatively associated with PTSS (*r *= –.21) (Zhai et al., 2015). Valentino et al. (2010) found that child-reported, but not parent-reported, hostile/coercive parenting was related to child-reported, and not parent-reported, PTSD severity (β = .38) (*N *= 91). Similarly, Goddard et al. (2019) found that child-reported (ρ =** **.36), but not parent-reported, critical parenting was associated with child-reported PTSS (*N *= 66).

Two further cross-sectional studies have specifically examined maternal and/or paternal parenting practices, with mixed results. Hendricks and Bornstein (2007) only explored maternal harsh parenting and found that child-reported maternal psychological control, but not rejection, was related to child-reported PTSS (β = .10) in 97 children exposed to the 9/11 attacks. El-Khodary and Samara (2019) found that child-reported paternal (*r* = .24) and child-reported maternal psychological control (*r* = .20) were each associated with child-reported PTSS, but not PTSD diagnosis, in 1029 children exposed to war.

We only identified a single longitudinal study in this area. Kelley et al. (2010) assessed 381 children following Hurricane Katrina. They found longitudinal associations between parent-reported corporal punishment at 3–7 months post-disaster and child-reported PTSS at both 3–7 months post-disaster (*r* = .16) and 13–17 months post-disaster (*r* = .17) (*N *= 381).

Overall, whilst there is tentative evidence for the relationship between harsh parenting practices and child PTSS, the majority of positive associations were based on child-reported parenting. Only a single study found evidence of such an effect based on parent reported harsh parenting and the association was small in magnitude (Kelley et al., 2010). The discrepant findings by informant could be due to inflation of effects due to single informant bias in the case of child reports, or limited willingness of parents to self-disclose or recognise harsh parenting behaviours. Conclusions are also limited by inadequate longitudinal evidence.

### Positive parenting

Fifteen studies explored the relationship between positive parenting practices and child PTSS (Table 1). Positive parenting refers to parenting practices characterised by adaptive social and emotional support for the child (see Table 2 for details). Six cross-sectional studies examined general aspects of positive parenting/support, with mixed results. Morris et al. (2016) found associations between child-reported positive parenting and child-reported PTSS following sibling death (*N *= 62) (*r* = −0.35). Thabet et al. (2009) found that higher child-reported parental support was associated with lower child-reported PTSS (β = –.33) and reduced prevalence of PTSD (*OR *= 0.96) in 412 war-exposed children. Bokszczanin (2008) found that child-reported parental support, specifically characterised by parental listening, financial and emotional support, showed a small inverse association with lower child-reported PTSS in a study of 533 children exposed to floods, (β = –.11). Conversely, El-Khodary and Samara (2019) found that child-reported maternal (but not paternal) support was positively associated with child-reported PTSS (β = .38), but not PTSD diagnosis in a second study of children exposed to war (*N *­  1029). Two further studies failed to find cross-sectional relationships between child-reported supportive parenting and child and parent-reported PTSS in children exposed to war (*N *= 211; Trentacosta et al., 2016) and to various trauma types (*N *= 91; Valentino et al., 2010).

In the only longitudinal study to examine broad elements of positive parenting, Dubow et al. (2012), found no longitudinal associations between positive parenting (non-physical strategies, reward/praise) at ages 8 and 11 and child-reported PTSS at 14 years old among 1501 children exposed to political violence.

Three studies specifically examined the role of parental emotional warmth and care, all using child-report to index both parenting and PTSS. Dekel and Solomon (2016) found a small, cross-sectional association between higher levels of maternal care and lower PTSS (*r* = –.04) in 2858 children exposed to war, but this effect did not survive adjustment for covariates (age, sex, prior trauma). Punamäki et al. (2001) found a negative association between paternal love and caring and child PTSS (β = –.36), but a cross-sectional positive association between maternal love and caring and PTSS levels (β = .35) in a longitudinal study of war-exposed children (*N *= 86). Finally, Gil-Rivas and Kilmer (2013), failed to find correlations between parent-reported warmth/acceptance reported 1-year post-disaster with child-reported 1 year or 2 years post-disaster Hurricane Katrina (*N *= 68).

Five studies examined reinstitution of roles and routines and child PTSS and found no evidence of an association, despite variation across studies in terms of whether child or parent reports were used. Prinstein et al. (1996) found no differences in parental maintenance of roles and routines across different PTSD severity levels in 506 children exposed to hurricanes. Four studies of injured children found no associations across cross-sectional (Williamson et al., 2019; *N *= 365) or longitudinal evidence (Hiller et al., 2018, *N *= 132; Marsac et al., 2013, *N *= 82; Marsac et al., 2014b, *N *= 243), including in a direct observation of parental encourage of approach coping (Hiller et al., 2018).

Overall, evidence for a relationship between positive parenting and child PTSS is limited. Some evidence was found for cross-sectional associations between general aspects of positive parenting and lower child PTSS but this was based entirely child reports, and both null findings and opposing effects were reported. No longitudinal associations were identified for any positive parenting domain. There was no evidence that parental reinstitution of roles and routines is related to child PTSD.

## Discussion

We reviewed the literature investigating the relationship between parental responses following a child’s traumatic event and child PTSS. We found relatively consistent of associations between child PTSS and parental overprotection, (lack of) trauma communication, trauma-related appraisals and encouragement of avoidance and distraction. There was mixed evidence for associations with harsh parenting and positive parenting. The evidence must be appraised carefully across all domains due limited evidence overall for some domains, limited longitudinal evidence, and use of single informants for child PTSS and parenting.

Relatively consistent evidence was found for associations between parental overprotection, avoidance of trauma discussion with the child and promotion of child distraction and avoidance of trauma-related stressors and child PTSS, albeit with mainly small effects. Each of these responses in the parent is likely to be a mechanism by which parents seek to limit their own distress and that of their child, particularly when child levels of distress are high, and to keep their child physically safe (Williamson et al., 2016; Williamson et al., 2019). Nonetheless, the evidence reviewed supports the conclusion that restricting children’s ability to engage with potentially challenging activities and emotions following trauma may have unintended negative consequences. If children are given limited opportunities to talk about or otherwise engage with trauma related thoughts and feelings, this could maintain maladaptive appraisals of the trauma and their reactions to it and impede the elaboration of the trauma memory, each of which has been linked to the maintenance of PTSD (Ehlers & Clark, 2000). More generally, parental overcontrol or limiting of children’s opportunities to act autonomously may negatively impact the child’s sense of self efficacy and maintain feelings of anxiety (Borelli et al., 2015).

The current findings suggest that addressing parental overprotection and (promotion of) avoidance could be one approach to facilitating children’s post-trauma recovery. However, the predictive value of these parental responses is still to be determined through further longitudinal studies. Further examination of parental communication is also important, as studies often relied upon brief questions to measure the *extent* of trauma talk, but the *nature* the caregiver-child discussions is also likely to be important (Salmon & Bryant, 2002). Research that simultaneously considers the amount and quality of trauma communication by parents is needed to provide a fuller understanding of their responses to child trauma.

It is more challenging to draw conclusions regarding the role of parental trauma-related appraisals and harsh parenting. Research consistently found parental trauma-related appraisals (e.g. about the child’s vulnerability and self-blame) to be associated with higher child PTSS across cross-sectional and longitudinal studies. If accurate, these findings are significant in identifying a modifiable risk factor with a clear potential underlying pathway – transmission of negative appraisals from parents to children, with consequent increased child PTSS (Hiller et al., 2018). However, only three studies explored parental appraisals. With respect to harsh parenting, positive associations were found between harsh parenting practices and child PTSS based on child reports, but not using parent reports. Future research can benefit from exploring these domains further and focusing on gathering information from both child and parent reports.

There was limited evidence for a relationship between positive parenting and lower child PTSS. Across ten studies examining general positive parenting or parental love/care. negative, positive and null associations with child PTSD were all reported. In addition, of the five studies that examined parental reinstitution of roles and routines, none found an association with child symptoms, despite cross-sectional and longitudinal designs with robust sample sizes. No longitudinal associations between positive elements of parenting and child PTSD were identified. Overall, these observations suggest that ‘negative’ parenting practices may yield larger or more robust effect sizes in relation to child PTSS compared to positive parenting. In intervention terms, promoting warm/positive parenting or parenting that facilitates a return to normal for the child may not be effective unless potential barriers to recovery (e.g. promotion of avoidance) are also addressed.

### Research limitations and recommendations

Overall, the quality of the included studies was mixed, especially in terms of the completeness of outcome data, study design and measurement of exposure. The findings from the current review should be interpreted considering the heterogeneity in the methodological quality of the studies. Ten studies had an unclear risk of bias due to incomplete outcome data. Seventeen of the studies reviewed used cross-sectional study designs, which limits the extent to which causational conclusions can be drawn. Where both cross-sectional and longitudinal effects were examined, cross-sectional effects typically failed to replicate longitudinally. Cobham and McDermott (2014) suggest that child PTSS are likely to be an important influence on how parents provide support, as well as the converse, resulting in a bi-directional relationship between parent and child post-trauma behaviours and psychological adjustment (Wilcoxon et al., 2021). Consequently, the dominance of cross-sectional data amongst the studies reviewed makes it difficult to determine the direction of the relationship between parenting behaviours and child PTSS or to isolate potential parental influences. In terms of measurement, 22 studies had a high risk of bias relating to single informants on child PTSS and parenting, and only one study used observational methods to study parent–child interactions. Studies also used highly varied measures of parental responses which made conflicting findings more challenging to interpret. Future research can take steps to reduce the risk of single-informant and self-report bias by collecting data from multiple informants on child PTSS and parenting responses, using the same measures more consistently across studies and also collecting observational data on parent–child interactions. This will facilitate stronger conclusions regarding the relationship between parenting and child PTSS.

There are also several limitations concerning the potential generalisability of the findings. There was limited evidence relating to children exposed to intentional traumas (e.g. physical or sexual assault), which carry higher risk of developing PTSD than non-intentional traumas such as disasters (Alisic et al., 2014), and we specifically excluded chronic/complex traumas and those potentially involving caregiving parent. Effects identified in the studies reviewed may not generalise to these groups. Second, certain ethnicities are overrepresented in the literature, meaning that children from minority ethnic backgrounds may not be adequately represented, and the majority of studies derived from the USA, Europe and Australia. The review was restricted to papers written only in the English language, which could have contributed to the limited representation of non-English speaking countries. This overrepresentation of certain countries and ethnicities is potentially significant, as the type of trauma exposure and trauma symptomatology may vary by race (Costello & Klein, 2019). Third, many of the included studies had an average age falling within late childhood to early adolescence and it is possible that any associations between parenting practices and symptoms may be reflective of an older sample, given obvious changes in parenting that occur through development. The possibility of moderation of parenting effects by child age should be considered in future research. Fourth, many studies had samples where parents/caregivers were entirely or mainly female. Therefore, findings may not be generalisable to male caregivers/fathers, which is important as El-Khodary and Samara (2019) and Punamäki et al. (2001) reported differences between maternal and paternal post-trauma parenting practices. Relatedly, although most studies had an equal split between male and female children, the potential influence of child sex was not investigated. Future research may benefit from exploring moderation by child and parent sex when investigating the relationship between parenting and child PTSS.

### Clinical implications

Research has most consistently suggested that parental overprotection, avoidance of trauma discussion and promotion of distraction and avoidance, are related to poorer outcomes in child PTSS. Potential therapeutic interventions could aim to teach parents more adaptive coping strategies to facilitate their child’s post-trauma recovery. There have been promising findings in involving parents in cognitive–behavioural therapies for child PTSD, which have included parental psychoeducation components informing parents about child PTSS and parenting practices (Hahn et al., 2019; Salloum et al., 2017). There have also been psychoeducational interventions targeting specific parental trauma -related responses, including trauma-specific parent–child communication and parenting coping strategies (Cobham et al., 2012; Haag et al., 2020; Marsac et al., 2019). Preliminary findings from these trials tentatively suggest that targeting parental responses, including parental overprotection and avoidance of trauma-related discussion, is beneficial for reducing child PTSS. However, evaluation of the long-term outcomes of these interventions is needed, especially as Haag et al. (2020) found comparable rates of child PTSS at 6-months when comparing a parenting intervention and treatment as usual. Future research could specifically target the longer-term effect of targeting trauma-related parenting behaviours, identify potential mediators/moderators of change, as well as the applicability of such interventions across different trauma populations.

## Conclusion

Parental overprotection, avoidance of trauma discussion, and promotion of avoidance and distraction in the child have relatively consistently been associated with poorer child PTSS outcomes. However, there is limited consistent longitudinal research, and the interpretation of results is often affected by informant bias. Notwithstanding these limitations, the findings from the current review suggest that future clinical interventions targeting these maladaptive parental responses could be one way to address children’s PTSS following traumatic exposure.

## Supplementary Material

Supplemental Material

Supplemental Material
