# Supplementary material for: A systematic literature review of the relationship between parenting responses and child post-traumatic stress symptoms
Source: Eur J Psychotraumatol. 2022 Dec 20;14(1):2156053. doi: 10.1080/20008066.2022.2156053 (PMC9788707; doi:10.1080/20008066.2022.2156053)
Supplement: Supplemental Material [file ZEPT_A_2156053_SM6191.docx]

| **Supplementary Material A**  *Summary of Included Studies* | | | | | | | | | | | |
| --- | --- | --- | --- | --- | --- | --- | --- | --- | --- | --- | --- |
| Reference | Study Design | Trauma type | Sample size | Child age in years, range and *M* (SD) | Parent age in years, *M* (SD) | Child sex | Parent sex | Child PTSS Measure | PTSS Informant | Parenting Informant | Parental response (measure) |
| Bokszczanin (2008) | Cross-sectional | Natural disaster (flood) | 533 children | Range not reported, 16.0 (2.50) | n/a | 60% female | n/a | Revised Civilian Mississippi PTSD Scale | Child | Child | Positive parenting, overprotection (unnamed measure^a^, Parental Bonding Instrument) |
| Carpenter et al. (2017) | Cross-sectional | Terror/bombing | 460 dyads | 4-19 years old, 11.8 (3.8) | 43.8 (7.8) | Not reported | 81% female | PTSD-RI | Parent | Parent | Trauma communication (study-specific questions^b^) |
| Cobham & McDermott (2014) | Cross-sectional | Natural disaster (minicylone) | 874 dyads | 8-12 years old, 9.71 (1.16) | Not reported | 55% female | Not reported | CTSQ | Child | Parent | Overprotection, trauma communication (study-specific questions) |
| Cohen & Eid (2007) | Cross-sectional | Terror/war-related | 346 children | 13-15 years old, *M* (SD) not reported | n/a | 46% female | 100% mothers | DISC-IV | Child | Child | Overprotection (study-specific questions) |
| Dekel & Solomon (2016) | Cross-sectional | War | 2858 children | 12-15 years old, 13.5 (0.65) | n/a | 53% female | n/a | CPTS-RI | Child | Child | Positive parenting, overprotection (Parental Bonding Instrument) |
| Dubow et al. (2012) | Longitudinal | Terror/political violence | 1501 parent-child dyads | 8-14 years old, mean not reported | Not reported | 51% female | 84% females | CPTSSI | Child | Parent | Positive parenting (CTSPC) |
| El-Khodary & Samar (2019) | Cross-sectional | War | 1029 children | 11-17 years old, 13.7 (1.36) | n/a | 52% female | n/a | PTSDSS | Child | Child | Positive parenting, harsh parenting (Parental Behavioural Inventory. PCS-YSR) |
| Felix et al. (2020) | Cross-sectional | Natural disaster (flood) | 485 parent-children dyads | 10-19 years old, 13.8 (2.56) | Not reported | 47% female | 69% female | CRIES-8 | Child | Both | Trauma communication (CRQ, unnamed scale^b^) |
| Garfin et al. (2014) | Cross-sectional | Natural disaster (earthquake) | 117 children | 7-9 years old, 7.59 (0.65) | n/a | 43% female | n/a | PTSD-RI | Child | Child | Caregiver-child conflict, trauma communication (Unnamed scale^c^) |
| Gil-Rivas & Kilmer (2013) | Longitudinal | Natural disaster (Hurricane Katrina) | Time 1: 68 caregiver-child dyads  Time 2: 53 caregiver-child dyads | Range not reported,8.5 (1.1) | 38.1 (9.6) | 56% female | 88% female | PTSD-RI | Child | Both | Positive parenting, coping strategies, trauma communication, parent appraisals, caregiver-child conflict (Unnamed scales^d, e, f^) |
| Goddard et al. (2019) | Cross-sectional | Various | 66 parent-child dyads | 8-17 years old, 13.5 (2.7) | 44.2 (6.7) | 41% female | 97% female | CPSS | Child | Both | Harsh parenting (BDSEE, PSEE, FQ) |
| Hendricks & Bornstein (2007) | Cross-sectional | Terror/9/11 | 97 mother-child dyads | Range not reported,13.9 (0.26) | 44.9 (4.68) | 44% female | 100% female | IES-R | Child | Child | Caregiver-child conflict, harsh parenting (CBQ, CRPBI) |
| Hiller et al (2018) | Longitudinal | Various | 132 parent-child dyads | 6-13 years old, 9.87 (1.8) | 39.7 (7.0) | 38% female | 90% female | PTSD-RI | Child | Parent | Overprotection, positive parenting, parent appraisal, coping strategies (PTRQ, POS) |
| Kelley et al. (2010) | Longitudinal | Natural disaster (Hurricane Katrina) | 381 parent-child dyads | 8-16 years old, 12.0 (2.0) | Not reported | Not reported | 99% female | PTSD-RI | Child | Parent | Coping strategies, parent appraisals, harsh parenting (Brief COPE, APQ, CRI) |
| Lavi et al. (2016) | Cross-sectional | War | 65 children | 11-18 years old, 12.3 (1.37) | n/a | 24% female | n/a | CPTS-RI | Child | Child | Trauma communication (Study-specific question) |
| Marsac et al. (2014) | Longitudinal | Motor vehicle accident | 243 parent-child dyads | 8-17 years old, 11.3 (2.5) | Not reported | 88% female | Not reported | CASQ | Child | Both | Positive parenting, coping strategies, parent appraisals (CCAC) |
| Marsac et al. (2013) | Longitudinal | Acute injury | 82 parent-child dyads | 8-17 years old, 12.1 (2.7) | Not reported | 30% female | 82% female | CPSS | Child | Both | Parent appraisals, positive parenting, coping strategies (CCAC) |
| Meiser-Stedman et al. (2006) | Longitudinal | Acute injury | 66 parent-child dyads | 10-16 years old, 13.8 (1.9) | Not reported | 39% female | 97% female | CRIES-13 | Child | Parent | Caregiver-child conflict, overprotection (FFQ) |
| Morris et al. (2016) | Cross-sectional | Death | 62 children, 88 parents | 8-18 years old, 13.0 (3.59) | Not reported | 65% female | 68% female | PTSD-RI | Child | Child | Positive parenting (APQ) |
| Prinstein et al. (1996) | Cross-sectional | Natural disaster (hurricane) | 506 children | 8-11 years old, M(SD) not reported; 3^rd^ grade = 32%, 4^th^ grade = 31%, 5^th^ grade = 37% | n/a | 56% female | n/a | PTSD-R | Child | Child | Trauma communication, parent appraisals, positive parenting, coping strategies (CCAC, SSSCA) |
| Punamäki et al. (2015) | Longitudinal | War | 240 children, 170 parents | 10-12 years old, 11.4 (0.57) | Mothers: 37.6 (6.92); fathers: 42.4 (7.75) | Not reported | Not reported | CRIES-R | Child | Parent | Harsh parenting (CSQ, SS) |
| Punamäki et al. (2001) | Longitudinal | War | 86 children | Range not reported,14.0 (0.79) | n/a | 51% female | n/a | CPTSD-RI | Child | Child | Positive parenting, harsh parenting (SPBIS) |
| Thabet et al. (2009) | Cross-sectional | War | 412 children | 12-16 years old, 13.7 (1.05) | n/a | 51% female | n/a | SCID | Child | Child | Positive parenting (PPSS) |
| Trentacosta et al. (2016) | Cross-sectional | War | 211 children | 8-22 years old, 12.8 (3.17) | n/a | 51% female | n/a | IES-R | Child | Child | Positive parenting (CYS items^g^) |
| Valentino et al. (2010) | Cross-sectional | Various | 91 children, 100 parents | 7-17 years old, 12.1 (2.9) | Not reported | 54% female | 89% female | PTSD-RI | Both | Both | Positive parenting, harsh parenting (Parent Behavior Inventory) |
| Williamson et al. (2018) | Cross-sectional | Various | 365 parent-child dyads | 2-19 years old, 8.2 (3.4) | Not reported | 45% female | 90% female | PTSD-RI | Parent | Parent | Parent appraisals, coping strategies, positive parenting, overprotection (POS, PTRQ) |
| Zhai et al. (2015) | Cross-sectional | Various | 5765 children | 12-18 years old, 12.5 (1.54) | n/a | 52% female | n/a | ETI-KJ | Child | Child | Harsh parenting (PAQ) |
| *Note.* ^a^4-item measure (Czapinski, 1998); ^b^3 items adapted from Afifi et al., 2008); ^c^7-item scale (Gil-Rivas et al., 2010; Greenberger & Chen, 1996); ^d^10-item scale (Greenberger & Chen, 1996); ^e^2 items (Gil-Rivas et al., 2007); ^f^20-tem scale (Gil-Rivas et al., 2007); ^g^3 items from the Communities that Care Youth Survey (Substance Abuse and Mental Health Services Administration, 2004)  CASQ = Child Acute Stress Questionnaire (Winston et al., 2002); CPSS = Child PTSD Symptom Scale (Foa et al., 2001); CRIES-8 = Children’s Revised Impact of Events Scale-8 (Yule, 1997); CRIES-R = Children’s Revised Impact of Events Scale (Dyregrov et al., 2002); CTSQ = Child Trauma Screening Questionnaire (Kenardy et al., 2006); ETI-KJ = Essen Trauma Inventory for Kids and Juveniles (Chinese Version) (Tagay et al., 2011; Zhou et al., 2010); IES-R = Impact of Events Scale – Revised (Weiss & Marmar, 1997); M-PTSD-R = Mississippi PTSD scale (Norris & Perilla, 1996); RIES-C = Revised Impact of Events Scale - Child Version (Dyregrov & Yule, 1995); SCID PTSD = Structured Clinical Interview for the DSM-IV Axis I Disorders, PTSD module (First et al., 1996); UCLA-PTSD-RI = UCLA PTSD Reaction Index (Pynoos et al., 1987; Frederick et al., 1992; Pynoos et al., 1998; Rodriguez et al., 1999; Steinberg et al., 2004)  APQ = The Alabama Parenting Questionnaire (Shelton et al., 1996); Brief APQ = Brief Alabama Parenting Questionnaire (Scott et al., 2011); Brief COPE = Brief Coping Orientation to Problems Experienced Inventory (Carver, 1997); BDSEE = Brief Dyadic Scale of Expressed Emotion (Medina-Pradas et al., 2011a); CCAC = The Children’s Coping Assistance Checklist (Prinstein et al., 1996); CBQ = Conflict Behaviour Questionnaire (Prinz et al, 1979); CRPBI = The Revised Children’s Report of Parental Behavior Inventory (Margolies & Weintraub, 1977); CRQ = Co-rumination Questionnaire (Rose, 2002); CSS= Child Informant Security Scale (Kerns et al., 2000); CSQ = Coping Strategies Questionnaire (Finnegan et al., 1996); SS= Security Scale (Kerns et al., 2000); CTSPC = Conflict Tactics Scales, Parent-Child Version (Straus et al., 1998); FFQ = Family Functioning Questionnaire (McFarlane et al., 1987); FQ = The Family Questionnaire (Wiedmann et al., 2002); PAQ = Parental Authority Questionnaire (Buri, 1991); Parent Behavior Inventory (Lovejoy et al., 1999); Parental Bonding Instrument (Parker et al., 1979); PCS-YSR = Psychological Control Scale–Youth Self-Report (Barber, 1996); POS = Parental Overprotection Scale (Edwards et al., 2010); PPSS = Perceived Parenting Support Scale (adapted from Mohammed, 1996); PSEE = Perceived Stress due to EE (Medina-Pradas et al., 2011b); PTRQ = Parental Trauma Response Questionnaire (Williamson et al., 2018); SSSCA = Social Support Scale for Children and Adolescents (Harter, 1985); SPBIS = Schaefer’s Parental Behaviour Inventory Scale (Schaefer, 1965) | | | | | | | | | | | |
