# Supplementary material for: A systematic literature review of the relationship between parenting responses and child post-traumatic stress symptoms
Source: Eur J Psychotraumatol. 2022 Dec 20;14(1):2156053. doi: 10.1080/20008066.2022.2156053 (PMC9788707; doi:10.1080/20008066.2022.2156053)
Supplement: Supplemental Material [file ZEPT_A_2156053_SM6134.docx]

| **Supplementary Material B**  *Risk of Bias Outcomes* | | | | | | | |
| --- | --- | --- | --- | --- | --- | --- | --- |
| Reference | 1.The selection of participants | 2. Confounding variables | 3. Measurement of exposure | 4. Study design | 5. Blinding of outcome assessments | 6. Incomplete outcome data | 7. Selective outcome reporting |
| Bokszczanin (2008) | Low | Low | High | High | Low | High | Low |
| Carpenter et al. (2017) | Low | Low | High | High | Low | Unclear | Low |
| Cobham & McDermott (2014) | Low | Low | High | High | Low | Low | Low |
| Cohen & Eid (2007) | Low | Low | High | High | Low | Unclear | High |
| Dekel & Solomon (2016) | Low | Low | High | High | Low | Low | Low |
| Dubow et al. (2012) | High | Low | High | Low | Unclear | Low | Low |
| El-Khodary & Samar (2019) | Low | Low | High | High | Low | Unclear | Low |
| Felix et al. (2020) | Low | Low | High | High | Low | Unclear | Low |
| Garfin et al. (2014) | Low | Low | High | High | Low | High | Low |
| Gil-Rivas & Kilmer (2013) | Low | Low | Low | Low | Unclear | Low | Low |
| Goddard et al. (2019) | Low | Low | Low | High | Low | Low | High |
| Hendricks & Bornstein (2007) | Low | Low | High | High | Low | Low | Low |
| Hiller et al (2018) | Low | Low | Low | Low | Low | High | Low |
| Kelley et al. (2010) | Low | Low | High | Low | Low | Unclear | Low |
| Lavi et al. (2016) | Low | Low | High | High | Low | Unclear | Low |
| Marsac et al. (2014) | Low | Low | High | Low | Low | Low | High |
| Marsac et al. (2013) | Low | Low | High | Low | Low | Low | High |
| Meiser-Stedman et al. (2006) | Low | High | High | Low | Low | Low | Low |
| Morris et al. (2016) | Low | Low | High | High | Low | Low | Low |
| Prinstein et al. (1996) | Low | Low | High | High | Low | Unclear | Low |
| Punamäki et al. (2015) | Low | Unclear | High | Low | Low | Low | Low |
| Punamäki et al. (2001) | Low | Low | High | Low | Low | Low | Low |
| Thabet et al. (2009) | Low | Low | High | High | Low | Unclear | Low |
| Trentacosta et al. (2016) | Low | Low | High | High | Low | Low | Low |
| Valentino et al. (2010) | Low | Low | Low | High | Low | Low | Low |
| Williamson et al. (2018) | Low | Low | High | Low | Low | Unclear | Low |
| Zhai et al. (2015) | Low | Low | High | High | Low | Unclear | Low |
